# Training of Brazilian Urology residents in laparoscopy: results of a national survey

**DOI:** 10.1590/S1677-5538.IBJU.2018.0668

**Published:** 2020-01-10

**Authors:** Wilson Francisco Schreiner Busato, Fernanda Girardi, Gilberto Laurino Almeida

**Affiliations:** 1 Universidade do Vale do Itajai ItajaíSC Brasil Disciplina de Urologia Universidade do Vale do Itajai - UNIVALI, Itajaí, SC, Brasil;; 2 Departamento de Uro-oncologia Sociedade Brasileira de Urologia Brasil Departamento de Uro-oncologia da Sociedade Brasileira de Urologia, Brasil;; 3 UroCenter CuritibaPR Brasil UroCenter, Curitiba, PR, Brasil

**Keywords:** Laparoscopy, Surgical Procedures, Operative, Training Support

## Abstract

**Objectives:**

To evaluate the familiarity of Brazilian urology residents with laparoscopy, methods of training and perspectives.

**Material and methods:**

a questionnaire with 23 questions was sent by e-mail to all urological residents of 86 Urology Residence Programs certified by the Brazilian Society of Urology (BSU).

**Results:**

225 valid answers (85% of all residents) responded. Most residences belong to academic hospitals mainly in the Southeast region of Brazil. Women account for 5% of residents and 82% of programs perform less than 100 procedures per year. Residents have access to LESS, RAL and 98% to surgical laparoscopy and 87% of these participate actively at the surgery, but 84.9% do not have access to RAL. The most common laparoscopic procedure is radical nephrectomy (73.2%), but only 28.8% of residents acted as surgeons, and third year residents (R3) are those that mainly performed this procedure (statistical significance, p <0.05). 61% of residents do not participate in hands-on courses or fellowship in laparoscopy, among those who attended these fellowships, 23.47% were sponsored by BSU in equal regions of the country. Although there are several opportunities of training in laparoscopy, 42% of residents do not have access to any kind of preparation and 52% have no structured specific program. R3 perception of laparoscopy experience is significantly higher than R2 and R1 residents. Almost 30% of them affirms that they are prepared for professional life regarding urologic laparoscopy.

**Conclusion:**

Brazilian urologic residents have access to laparoscopy and actively participate in the learning process. Robotic surgery is expanding in the country, although still very far from residents. Brazilian resident, at the end of medical residency, is motivated to perform laparoscopic procedures.

## INTRODUCTION

Over the last 20 years, laparoscopic surgery has gained popularity in Urology, reducing morbidity, convalescence period and with improving results, and has become the gold standard treatment of many urologic diseases (1-3). However, until nowadays training in laparoscopy is challenging in underdeveloped countries, frequently, public health services do not provide this treatment and availability of experienced urologist is insufficient (4, 5). With consolidation, more complex procedures were incorporated, and Urology Residence Programs (URP) could not accompany this evolution. Part of the problem is caused by the learning process based on Halsted (1889): “see one, do one, teach one”. This aphorism is very useful to open surgery, but for laparoscopy is inadequate, since the procedure requires acquisition of skills, and a more step-by-step model must be used. This is challenging to most Brazilian URPs (6, 7). There are several evidences that an adequate training helps achieving proficiency in laparoscopy (6-8). Therefore, it is ideal that a residence program contains a well-structured learning skill program in urologic laparoscopy, that performs most of available procedures (3, 9, 10).

In Brazil, most urology residents do not have access to laparoscopic training (11). Therefore, it is important to know the reality of training in urologic laparoscopy among Brazilian residents in order to improve and develop resources for better training (1).

The objective of the present study is to evaluate the access of Brazilian urology residents to laparoscopy surgery and the pattern of training in urologic laparoscopy.

## PARTICIPANTS AND METHODS

A question form containing 23 questions was proposed, based on the study of Furriel et al. (7). After review of the Teaching and Learning Comission of Brazilian Society of Urology (TLC-BSU), the questionnaire was published in an online questionnaire specialized website ( SurveyMonkey™, Palo Alto, USA) that was sent by email to all residents of the 86 Residence in Urology Programs (URP) certified by BSU. The question forms were sent again three times every 15 days. It was established a period of 6 months (July 2016 to January 2017) for answer.

The questionnaire evaluated five main aspects: i. Personal and professional characteristics of residents: ii: resident access to laparoscopy, iii: resident experience with laparoscopy, iv: training in laparoscopy and v: motivation and future perspectives.

The answers were included in a database and analyzed by descriptive statistical methods using IBM SPSS Statistics Version 21.0 software (IBM, New York, USA). X^2^ test was used to compare quantitative variables among groups. P <0.05 was considered statistical significant.

## RESULTS

Among 265 residents of all URP certified by BSU at the time of the study, 225 (85%) answered the questionnaire.

### Personal and professional characteristics of Urology residents

Demographic data are presented at Table-1. Women are less than 5% of residents, most are from Southeast region and in academic hospitals (68%). Almost half of responders (48.9%) are last year residents and 16% of first year. Around 82% of URPs perform less than 100 laparoscopic procedures/year for resident training and 5% more than 250 procedures/year.

**Table 1:** Demographics and services characterization.

Variable	Frequency	%
**Sex**		
Male	212	95,5%
Female	10	4,5%
No reply	3	
**Age years**		
26-30	109	49,1%
31-35	105	47,3%
36-40	5	2,3%
>40	3	1,4%
No reply	3	
**Hospital Type**		
Academic	151	68,0%
Non-academic public	45	20,3%
Private	26	11,7%
No reply	3	
**No. of PRU residents**		
1-3	52	23,3%
4-6	112	50,2%
7-9	39	17,5%
>9	20	9,0%
No reply	2	
**What is your year of residence in Urology? (Outside General Surgery)**		
1st year	36	16%
2nd year	77	34,2%
3rd year	110	48,9%
No reply	2	
**How many urological laparoscopic procedures are performed on the PRU per year?**		
None	2	0,9%
1-50	94	42,3%
51-100	88	39,6%
101-250	27	12,2%
>250	11	5,0%
No reply	3	

### Access of residents to laparoscopy

**Figure 1:**
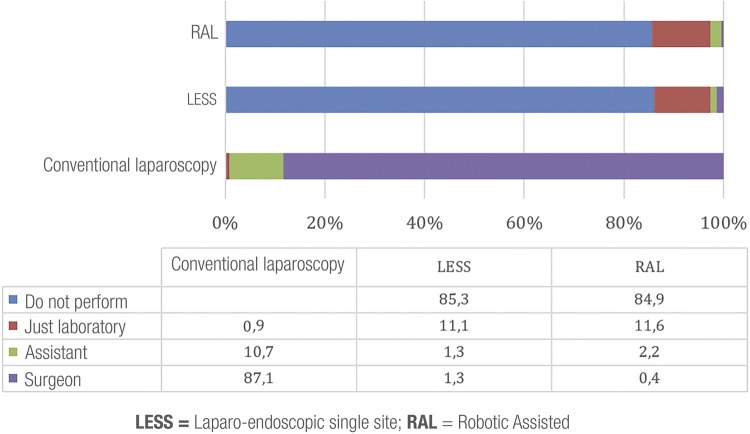
Participation of residents in surgeries.

Brazilian residents follow different surgical techniques such as conventional laparoscopy, single site laparoscopy (LESS) and robotic surgery (RAL), a sub-analysis of laparoscopic techniques showed that almost all residents (98%) accompany conventional laparoscopy and only one resident answered that did not have access to laparoscopy (Figure-1). Considering this URPs characteristic, residents are able to participate in laparoscopic procedures as auxiliary surgeon or as first surgeon in up to 87% of procedures. Also, 85.3% and 84.9% of residents have no access to LESS and RAL, respectively.

### Experience of residentes in laparoscopy

Residents were asked about their experience as surgeons in specific procedures (Table-2). Most performed laparoscopy procedure was radical nephrectomy (73.2%) but only 28.8% acted as surgeon. Next, it was informed pyeloplasty (56.8%) and marsupialization of renal cyst (54.4%). Less performed surgeries included radical cystectomy, probably due to its high complexity, and sacrum-promontory-fixation. Procedures that require higher skills such as partial nephrectomy and radical prostatectomy were not performed by 56.7% and 69.9% of residents, respectively.

**Table 2:** Laparoscopic surgeries performed by residents as a surgeon.

	Residents		

Procedure	Number of procedures	Frequency	%
**Total nephrectomy**	None	43	26.9
	1 a 10	71	44.4
	> 10	46	28.8
**Partial Nephrectomy**	None	80	56.7
	1 a 10	57	40.4
	> 10	4	2.8
**Renal cryoablation**	None	107	93.0
	1 a 10	8	7.0
**Pyeloplasty**	None	63	41.4
	1 a 10	86	56.6
	> 10	3	2.0
**Adrenalectomy**	None	79	56.4
	1 a 10	61	43.6
**Renal cyst marsupialization**	None	68	45.6
	1 a 10	79	53.0
	> 10	2	1.3
**Radical prostatectomy**	None	93	69.9
	1 a 10	28	21.1
	> 10	12	9.0
**Radical cystectomy**	None	108	90.8
	1 a 10	10	8.4
	> 10	1	0.8
**Promonto-fixation**	None	100	85.5
	1 a 10	16	13.7
	> 10	1	0.9
**Varicocelectomy**	None	98	79.0
	1 a 10	23	18.5
	> 10	3	2.4
**Orchidopexy**	None	90	66.2
	1 a 10	41	30.1
	> 10	5	3.7
**Lithiasis surgery**	None	64	43.2
	1 a 10	61	41.2
	> 10	23	15.5

According to year of residency, when residents performed laparoscopic procedures as first surgeons (Table-3), third year residents are the most frequent to perform radical nephrectomy as first surgeon, with statistical significance (p <0.05). If we consider marsupialization of renal cyst, there are no statistical difference between R3 and R2, although significantly different of R1.

**Table 3:** – Year of residence in which the resident performs the procedure as a surgeon.

	Year of first procedure			

	1st year	2nd year	3rd year	Responders
Total nephrectomy	5.8%	33.1%	61.2%	103
Partial Nephrectomy	6.0%	22.0%	72.0%	50
Renal cryoablation	50.0%	12.5%	37.5%	8
Pyeloplasty	7.9%	21%	70.1%	76
Adrenalectomy	5.9%	15.7%	78.4%	51
Renal cyst marsupialization	12.9%	37.2%	50%	70
Radical prostatectomy	-	31.2%	68.8%	32
Radical cystectomy	-	20.0%	80.0%	10
Promonto-fixation	17.6%	35.3%	47.1%	17
Varicocelectomy	34.8%	30.4%	34.8%	23
Orchidopexy	23.1%	30.7%	46.2%	39
Lithiasis surgery	11.6%	34.8%	53.6%	69

### Laparoscopic Training of Residents

**Figure 2:**
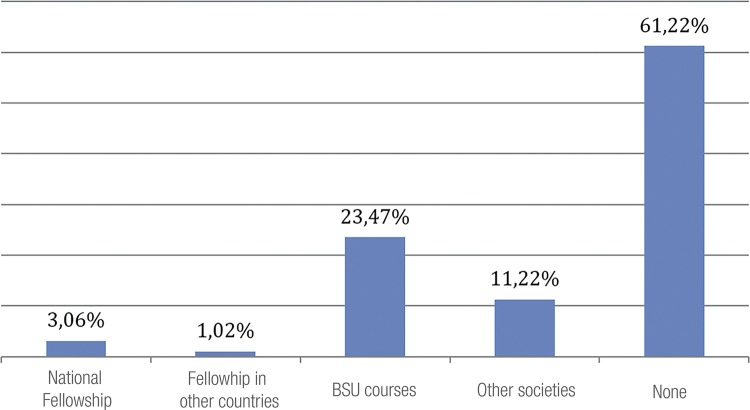
Hands on or fellowship in urologic laparoscopy.

The study asked residents about the available opportunities to attend hands on or fellowship in urologic laparoscopy. As shown in Figure-2. 61% of residents do not participate in any training during their URP. Among those who attended, 23.47% are promoted by BSU and it is important to draw attention to the fact that 11.22% attended those trainings promoted by other medical societies.

When they were asked about the structure of their services, 52% of residents responded that there was no specific laparoscopy training program during residence.

**Figure 3:**
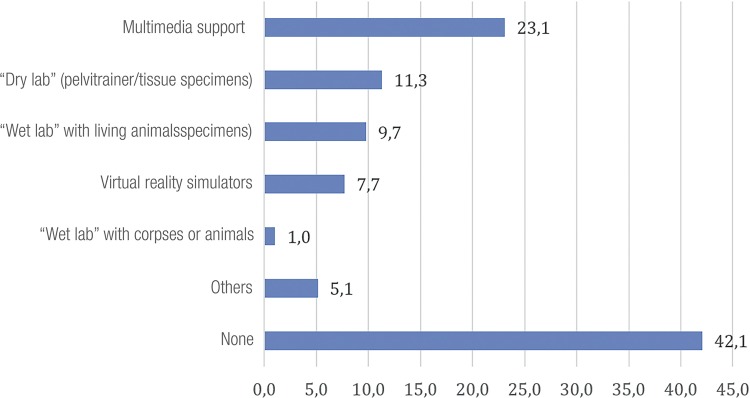
Available trainings at residence.

Most opportunities of training are parted, as shown in Figure-3, however, almost 42% of residents have no access to any training in laparoscopy.

When analyzed according to the region of Brazil, there is no statistical difference among residents that attend BSU courses, demonstrating an equal distribution of trainings among different regions of Brazil (Table-4).

**Table 4:** Participation of residents distributed by region.

		Region				
	Total	North	Northeast	Midwest	Southeast	South
Fellowship in national hospital	3.1%	0.0%	2.6%	5.6%	3.6%	0.0%
Fellowship other countries Hospital	1.0%	16.7%	0.0%	0.0%	0.9%	0.0%
Course promoted by SBU	23.5%	33.3%	25.6%	22.2%	24.1%	14.3%
Course promoted by other societies	11.2%	0.0%	12.8%	11.1%	10.7%	14.3%
I never attended a course or fellowship	61.2%	50.0%	59.0%	61.1%	60.7%	71.4%

### Motivation and future perspectives

Residents were also questioned about their current laparoscopic experience (at the time of questionnaire) (Figure-4a). R3 perception, as expected, had more answers of “satisfactory”, ‘good” and “very good”, significantly different of R2 and R1s. However, at the end of residence, 15.5% of R3 answered “very poor” or “poor” (21.6%), indicating that 37.1% judged themselves unprepared for urologic laparoscopy.

**Figure 4a:**
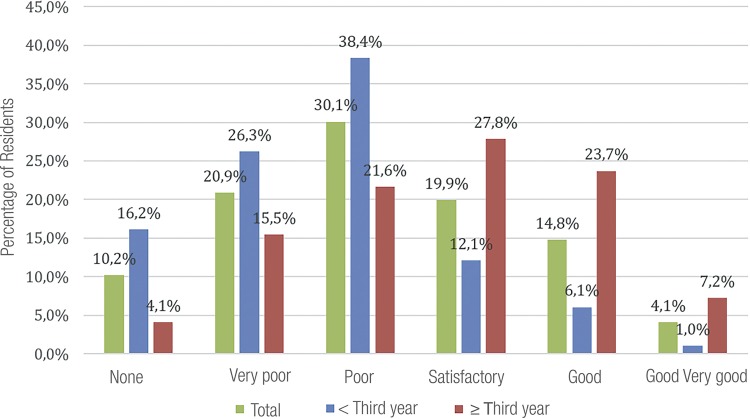
Laparoscopic experience at the time of questionnaire.

Finally, they were asked about their expectations of laparoscopic experience at the end of residence (Figure-4b). It is observed that there are no statistical differences between R1 and R2, and R3. Both groups have good expectations at the end of residence.

**Figure 4b:**
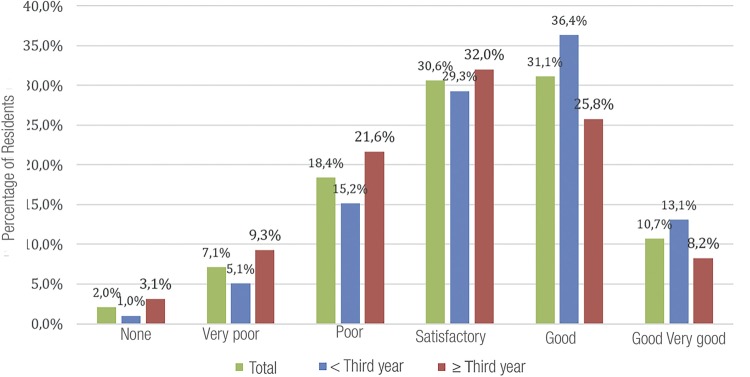
Expectations of laparoscopic experience at the end of residence.

## DISCUSSION

In order to change Brazilian urology, to incorporate new technologies, it is important to invest in medical learning in Brazilian URPs. It is challenging to introduce laparoscopic training in a poor underdeveloped country,, particularly in public health services URPs. The need is overwhelming, as demonstrated by the high answer of Brazilian residents (85%), compared to Italy (72.6%), Portugal (71.6%), Spain (68%), Germany (65%) and only 3.1% in European Community (1, 7, 12).

It was shown that there is a higher concentration of residents in the southeast region of Brazil. That region presents the higher demographic density and higher concentration of hospitals and teaching institutions (13). As in Europe, the higher number of residents belong to academic institutions (68%), reinforcing the teaching bias of medical residence (7). Mean age of residents was 26 to 30 years, mainly males. In Italy and Portugal, 22% and 13% respectively are female (1, 14), while in Brazil only 4.5% are female. Otherwise, in Germany, most urology residents are female (55%) (15).

The amount of laparoscopic training (access to laparoscopy training offered by URP) reflects the future integration of the professional with advanced laparoscopic skills, characteristic of the specialty (16-18). Our results indicate that most residents (98%) have access to laparoscopy, but only 87% participate as surgeon. Therefore, in 12% of URPs there are laparoscopic surgeries but not training, the residents act only as auxiliary surgeons and only 1% attend only laboratory. This difference is related to the fact that the preceptor has a learning curve (institutional learning) and/or the preceptor is responsible for the patient and not the URP (11). In Europe, 78.8% of residents have access to laparoscopy, 56% in Germany, 25.8% in Italy and 100% in USA (1, 16, 12). 14.7% and 15.1% of Brazilian residents have access to most recent technologies such as LESS and RAL, similar to 17% of European residents (7). In USA and Canada, countries with a great number of platforms, 54% and 36% have robotic training (16, 19). It is important to stress that in the year when the questions forms were applied, in Brazil there were only 10 da Vinci® platforms; at present, there are 41 robotic platforms with a perspective of 15% of year growth (industry data).

On the other hand, Brazilian residents act more as surgeons (87%) if compared to European residents (27%) and Portuguese residents (32.7%) (1, 7). This huge difference may be related to the methodology of the question form, that may be interpreted differently in the different countries of European Community and lack of clear definition of which participation percentage as surgeon is related to this denomination. Another possible explanation is that we reproduce the teaching methodology of open surgery, where “doing” is sooner (“see one, do one, teach one”).

General surgery residence (GS) initiates surgical learning, including laparoscopy, and is mandatory for urologic training, At present, most Brazilian GS residences include training in initial laparoscopy (11). Only 6.3% of residents answered that they had no training in laparoscopy during general surgery residence, but among those 93% that experienced it, 26% indicated a bad training. The others evaluated positively their laparoscopic preparation before joining an urology residence. Therefore, almost one third (27.7%) of residents that joined the URP have an inadequate initial laparoscopic training. Those must be identified and offered complementary training to overcome this deficiency to not jeopardize the urology laparoscopic training. Hands on courses and immersion programs may help this task.

This study shows that the main form of teaching in Brazil is still direct training in patients of URP programs, under the supervision of preceptors, as observed in other studies (20), therefore, the learning curve of most urologic procedures is slower, since it is not possible to have an equal number of procedures (21, 22), and the number of laparoscopic procedures is very important to acquisition of skills. It is worrisome that 42.3% of URPs of Brazil do not perform more that 50 laparoscopic procedures/year. In 82%, less than 100 procedures/year. Also, it must be taken in consideration that there are at least two residents (R2 and R3) to share laparoscopic procedures and that only 30% of URPs have 1 resident/year, 48.8% have 2 residents/year, 11.2% have 3 residents/year, and there are residences with up to 8 residents/year (BSU Teaching and Learning Commission data). Training must include several procedures. Borgmann et al. referred that in order to increase exposition of residents to laparoscopy, the best way is to reduce the number of residents/year (15).

The time of access to learning is important during resident training (22). That study indicated that 33%, 34% and 33% of residents have experience as first urology laparoscopy surgeon during R1, R2 and R3, respectively. This equal distribution means a priori that a step-by-step approach is being implemented at the URPs in relation to laparoscopic training. Two other aspects may explain this high number during first year of residence: they finish GS training with adequate skills and incorporated less complex procedures to laparoscopic techniques.

The most performed procedure, as in the European study, was laparoscopic radical nephrectomy (LRN), since this procedure is the gold standard for the treatment of renal tumors, when it is not possible to perform partial nephrectomy (PN) (2). This information may direct URPs to which procedure adopt to initiate laparoscopic training. In Brazil, LRN is performed by 61.2% and 33.1% of R3 and R2, respectively. This may raise a discussion about if LRN may be performed by R2 as prerequisite for R3 LPN. In Brazil, 73.2% of residents perform at least one LPN during their residence, in contrast to one third of European residents (7).

In sequence, the most performed procedures are pyeloplasty, marsupialization of renal cyst, and stone laparoscopic surgery. A possible explanation is that these are abdominal surgeries similar to those learned during general surgery residence. In this context, more difficult technical procedures, specially pelvic surgeries, such as radical prostatectomy and cystectomy are performed by less number of residents. Also, partial nephrectomy is not performed by more than 50% of residents. This fact may be explained that this is a complex procedure and was recently considered gold standard for T1 and T2 tumors (2).

Ideally, training programs must develop several basic skills in order to the residents fell comfortable to perform laparoscopy in their subsequent practice (9, 6). In Brazil, more than 80% of URP do not evaluate proficiency in laparoscopy before surgery in humans and less than half of services have a specific teaching laparoscopy program (5). These data corroborate the difficulty of URPs to change the teaching methodology to a step-by-step approach, maintaining the traditional Halsted approach for open surgery.

An alternative for this change of teaching paradigm is training in simulator laboratory. It improves significantly the resident skills and is associated to several advantages, including speed and quality, particularly in this initial phase of training in special if present in the URP itself (3, 6, 9, 22-24). In our study, 42% of URPs do not have any training facility for laparoscopy. In Portugal, it is observed in 35.7% of URPs and in 41.7% of European Community URPs (1, 7).

A little more than 20% have a video database, drylab (11%), wetlab (9%) and virtual reality simulators (7%). Even when available, 76% of support laboratories are not structured to teach programs. The final objective of a simulator program is to show the possibility of transference of acquired skills at the laboratory for the clinical scenario, allowing for objective quantification of operational clinical performance following the simulator training (24).

In a recent analysis about laparoscopic training in GS residences in Brazil, Nácul et al. (11) affirmed that the failure of a good professional training is related to the lack of a pedagogic model based on intensive courses. It not provides surgical experience for an adequate and safe laparoscopic performance. This modality, that should have been used for punctual situations, is been used for replacement of insufficient training (25).

The lack of a facility is related that most residents never attended to hands on or fellowship laparoscopic courses, but most answers were pointed by R1 and R2 residents. Those who attended any course were sponsored by Brazilian Society of Urology and most were R3. Most residents, particularly R3, are motivated to perform laparoscopic surgeries at the end of the course. In this scenario, 60% of residents intend to join a fellowship at the end of residence, in order to complement training with more advanced procedures/techniques or as a sign of unprepared training.

Teaching process premises a constant feedback with the student (22). Most residents negatively evaluate their current experience in laparoscopy. This initial worrisome fact is observed in all residents, including R1, that are initiating their laparoscopic urological training. However, according to the year of residence, R3 are more positive, indicating confidence and skill gain. At the end of the course, 72% had a positive estimative.

One limitation of the present study is that the questionnaire was responded by all residents and not only by R3, underestimating the skills. Most responders were first year residents indicating a bias on the performance of laparoscopy. However, it is important to remind that those same residents will have opportunities to improve their skills over residence. Another aspect is that all answers were individual and subjected to individual interpretation, the differences among Brazilian regions difficulted an homogeneous question form answer.

## CONCLUSIONS

Urologic residents in Brazil, in general, have access to laparoscopy and participate actively in the learning program, although with a limited number of procedures. With the expansion of robotic surgery in Brazil, and the inevitable replacement as main surgical modality, it is expected that residence programs also include this new reality in their teaching programs.

Although there are pedagogic errors based on the old model of learning or surgical techniques, with the endorsement of Brazilian Society of Urology and other medical societies, that incentive the attendance to preparatory courses of laparoscopic surgeries, the Brazilian resident, at the end of his program, is motivated to perform laparoscopic procedures.
